# Comprehensive Genomic and Proteomic Analysis Identifies Effectors of *Fusarium oxysporum* f. sp. *melongenae*

**DOI:** 10.3390/jof10120828

**Published:** 2024-11-28

**Authors:** Jiayelu Wu, Pengfei Wang, Wuhong Wang, Haijiao Hu, Qingzhen Wei, Chonglai Bao, Yaqin Yan

**Affiliations:** 1 Institute of Vegetable, Zhejiang Academy of Agricultural Science, Hangzhou 310021, China; 2 Zhejiang Normal University, Jinhua 321004, China

**Keywords:** effector proteins, *F. oxysporum* f. sp. *melongenae*, bioinformatic prediction, LC-MS

## Abstract

*Fusarium* wilt in eggplant caused by *F. oxysporum* f. sp. *melongenae* is a major devastating soil-borne disease on a worldwide scale. Effectors play important roles in the interactions in pathogen–plant interactions. Identifying effectors is essential for elucidating the pathogenic mechanisms of phytopathogenic fungi. In this study, bioinformatic prediction approaches, including SignalP v5.0, TMHMM v2.0, WoLF PSORT, PredGPI, and EffectorP, were employed to screen for candidate secreted effector proteins (CSEPs) in *F. oxysporum* f. sp. *melongenae*. A total of 1019 proteins exhibiting characteristics typical of classical secretory proteins were identified, 301 of which demonstrated carbohydrate activity, and 194 CSEPs were identified. Furthermore, a total of 563 proteins from *F. oxysporum* f. sp. *melongenae* under induced conditions were identified using mass spectrometry-based label-free quantitative proteomics. These findings suggest a potential role of these CSEPs in the interaction between *F. oxysporum* f. sp. *melongenae* and eggplant, thereby contributing to a deeper understanding of the pathogenic mechanisms of *F. oxysporum* f. sp. *melongenae* and strategies for disease management.

## 1. Introduction

*Fusarium oxysporum* is an important phytopathogen with a broad host range, infecting both dicots and monocots and causing wilt in many economically crucial crops, such as eggplant, bean, and tomato [1]. *F. oxysporum* causes wilting in roots and hypocotyl cortical tissues and moves towards the vascular bundle through intercellular spaces without reaching the vascular system [2]. However, when a singular strain exhibits a specialized infestation of different host plants, the strains which have the same host range can be subdivided into a physiological microspecies. The *F. oxysporum* species complex (FOSC) is separated into several groups or formae specialis; for example, the strain that specifically infects eggplant is classified as *F. oxysporum* f. sp. *melongenae* (Fom), the strain that specifically infects tomato is classified as *F. oxysporum* f. sp. *lycopersici* (Fol), the strain that infects chickpea is classified as *F. oxysporum* f. sp. *ciceri* (Foc), and *F. oxysporum* f. sp. *pisi* (Fop) specifically infects pea. For the development of durable crop control strategies, it is crucial to gain a deeper understanding of the mechanisms of the interactions between diverse *F. oxysporum* and their hosts. In the interaction between plants and pathogens, plants detect pathogen-associated molecular patterns (PAMPs) and damage-associated molecular patterns (DAMPs) through pattern recognition receptors (PRRs) located in the plasma membrane, thereby activating a primary defense mechanism referred to as PAMP-triggered immunity (PTI) [3]. Fungal effector proteins play an important role in these processes, which can increase host susceptibility via multiple pathways [4]. For example, the effector protein Cmu1 from *Ustilago maydis* decreases the accumulation of the salicylic acid precursor chorismate, thereby inhibiting host ETI [5]. The effector PlAvh142 in *Peronophythora litchi* plays a crucial function in the induction of cell death in host plants [6], and the Avr1 effector from *F. oxysporum* f. sp. *lycopersici* enhances pathogenicity by repressing resistance mediated by I-2 and I-3 R proteins [7].

The absence of conserved sequences among known fungal effectors makes the large-scale identification of putative fungal effectors remain challenging, and thus far, only a limited number of pathogenic fungal effectors have been isolated and analyzed for their functions. Nonetheless, advancements in whole genome sequencing have facilitated the computational prediction of effector proteins. This rapid and cost-effective approach allows for the identification of CSEPs, which can be experimentally validated to enhance our comprehension of the molecular dynamics between plants and pathogens [8]. Many studies have shown that effectors have the same typical characteristics to accurately predict CSEPs [9,10,11]: (i) having N-terminal signal peptides; (ii) consisting of less of 300 amino acid residues; (iii) lacking transmembrane domains; (iv) predicting location signals for protein delivery to extracellular regions; and (v) lacking GPI-anchor sites. Based on these characteristics, several studies have harnessed the power of computational methods to predict the existence of CSEPs in various pathogenic fungi. For example, there has been the identification of 725 CSEPs in the flax rust pathogen *Melampsora lini* [8]; the detection of 78 CSEPs in the necrotrophic plant pathogen *Sclerotinia sclerotiorum* [12]; the discovery of 80 CSEPs in *Leptosphaeria maculans* [13]; and the prediction of 32 CSEPs in *Plasmodiophora brassicae* [14].

*Fusarium* wilt, caused by *F oxysporum* f. sp. *melongenae*, is a significant and destructive soilborne disease affecting eggplant worldwide. The primary strategy for managing *Fusarium* wilt in eggplant has been the selection of resistant varieties. While resistance linked to quantitative trait loci has been documented, gene-for-gene interactions within the *F. oxysporum* f. sp. *Melongenae*–eggplant pathosystem remain uncharacterized [15,16]. To date, limited pathogenicity/virulence factors have been reported for *F. oxysporum* f. sp. *melongenae*. In this study, we employed a variety of computational prediction methods, such as SignalP, WoLF PSORT, TMHMM, and EffectorP to predict CSEPs. Despite the strategic efficiency of software in predicting effector proteins, it is vital to undertake experimental verification to delve into the specifics of plant–pathogen interactions. We conducted a functional validation of the predicted signal peptides using a yeast signal sequence trap system and investigated gene function via an *A. tumefaciens*-mediated transient expression assay. Our findings provide a basis for identifying effector proteins from *F. oxysporum* f. sp. *melongenae*, thereby enhancing the understanding of the molecular mechanisms governing plant–pathogen interactions.

## 2. Materials and Methods

### 2.1. Plant Materials and Fungal Isolates

The *F. oxysporum* f. sp. *melongenae* used in this study was the FOM-1 strain which was obtained from Hebei Agricultural University and cultured on potato dextrose agar (PDA) at 25 °C under dark conditions. The eggplant variety E25 was maintained in our laboratory, while *N. benthamiana* was grown in a greenhouse under a 16/8 h light/dark photoperiod at 25 °C. Competent *Escherichia coli* DH5α cells were employed for plasmid amplification, and the *A. tumefaciens* strain GV3101 (Shanghai Weidi Biotechnology Co., Ltd., Shanghai, China) was utilized for conducting transient gene expression through *A. tumefaciens*-mediated methods.

### 2.2. Prediction of Fungal CSEPs and Core Effectors

The complete genome sequence of Fom was previously acquired from the JGI database (JGI Genome Portal-Home (accessed on 21 November 2023)). SignalP v5.0 was employed to detect N-terminal signal peptides and their cleavage sites [17]. Then, TMHMM 2.0 was used to predict the transmembrane domain in these sequences with default parameters. WoLF PSORT was used to predict subcellular locations. PredGPI was used to find and exclude ankyrin sequences. As result, a proteome with classic secreted protein signatures was obtained. SecretomeP 2.0 Server was used to screen proteins that did not contain N-terminal signal peptides and had an NN-score > 0.5 to obtain nonclassical secreted proteins.

To further validate the obtained secretory proteome, TargetP 2. 0 was used to ensure that they contained signal peptides and could be secreted extracellularly. Since most of the fungal CSEPs were <300 amino acid residues in length and cysteine-rich, only a few of the larger proteins were also identified as fungal effector proteins. Therefore, the sequence with cysteine ≥ 4 and amino acid lengths less than 300 were obtained using seqkit software v2.5. EffectorP 2.0 was used for effector prediction. The PHI database was used for blasting and pathogenic gene analysis, the parameters were set to E-value = 1.0 × 10^−10^, and the num alignments were set to 100.

### 2.3. Mass Spectrometry-Based Label-Free Quantitative Proteomics Analysis of Secreted Proteins from the Fom

The FOM-1 strain was cultured on potato dextrose agar (PDA) medium for 7 d. Subsequently, 0.5 cm squares of mycelial agar blocks were taken with sterile toothpicks and transferred to potato dextrose broth (PDB) medium. The culture was incubated at 28 °C with shaking at 200 rpm for 5 d, followed by centrifugation at 5000 rpm for 10 min. The supernatant was discarded, and the resulting pellet was resuspended in sterile water to an optical density (OD_600_) of 0.8–1.0. Eggplants at the 2–4 leaf stage was washed to remove the soil from the roots. Half of the roots were excised, and the plants were immersed in FOM-1 spores for 5 d. The infiltrate was then centrifuged at 12,000 rpm for 10 min, and the supernatant was collected and concentrated using ultrafiltration with a membrane with a molecular weight cut-off (MWCO) of 10 kDa. The proteins in the solution were precipitated utilizing the acetone precipitation method.

LC-MS/MS analysis was performed utilizing a Q Exactive mass spectrometer (Thermo Scientific, Shanghai, China) paired with an Easy nLC system (Proxeon, now Thermo Fisher Scientific, Shanghai, China) over periods of 60, 120, and 240 min. Peptides were preconcentrated on a Thermo Scientific Acclaim PepMap100 reverse-phase trap column (100 μm × 2 cm, nanoViper C18) before the analysis on a C18 analytical column (Thermo Scientific Easy Column, 10 cm × 75 μm, 3 μm resin). The mass spectrometer operated in a positive ion mode and employed a data-dependent top10 approach for precursor ion selection from survey scans covering the range of 300–1800 *m*/*z* and was subsequently subjected to HCD fragmentation. The AGC target was set to 3.0 × 10^−6^, with a maximum injection time of 10 ms and a dynamic exclusion period of 40 s. The survey scans were acquired at a resolution of 70,000 (*m*/*z* 200), while the HCD spectra were recorded at 17,500 resolutions using a 2 *m*/*z* isolation width. Normalized collision energy was maintained at 30 eV, alongside a 0.1% underfill ratio and an activated peptide recognition mode. The obtained LC-MS/MS data were searched with MaxQuant software (version 1.5.3.17) against the Fom genome (GCA_001888865.1). The protein FDR and peptide FDR were less than 1%. Unique peptides were used for protein identification. The proteins of each biological repetition with a peptide coverage of more than 20% were set as reliable.

### 2.4. Plasmid Construction and Preparation

Total RNA was extracted from the Fom-1 strains with an RNAsimple isolation kit (Tiangen, Beijing, China). The cDNA synthesis was performed using HiScript II Q RT SuperMix (Vazyme, Nanjing, China). CSEPs were cloned from the cDNA of the FOM-1 strains utilizing a 2× Phanta Max Master Mix (Vazyme, Nanjing, China). The BamHI restriction site was incorporated into the PVX vector, followed by the ligation of the genes with the vector using the ClonExpress Ultra One Step Cloning Kit (Vazyme, Nanjing, China). The primers used for the plasmid construction are listed in Supplementary Table S1.

### 2.5. Transient Expression of Target CSEPs in N. benthamiana

The *Agrobacterium*-mediated transient expression in *N. benthamiana* was conducted following previously established protocols [18]. Briefly, *Agrobacterium* cultures were cultivated in LB medium to reach an absorbance of 0.6 at OD_600_. Cells were harvested via centrifugation at 5000 rpm for 10 min and then resuspended in MgCl_2_ buffer (10 mM MgCl_2_, 10 mM 2-morpholinoethanesulfonic acid monohydrate, 200 µM acetosyringone) to obtain an optical density of 0.8 at OD_600_. Six-week-old *N. benthamiana* leaves were employed for agroinfiltration assays, and a needleless syringe was utilized to deliver the aliquots of bacterial suspensions. To evaluate cell death suppression, the leaves were infiltrated with an *A. tumefaciens* strain carrying a plasmid that expresses BAX, and subsequent infiltration with a strain containing CSEPs followed after 24 h. The *A. tumefaciens* transformed with GFP and BAX were used as negative controls and positive controls, respectively.

### 2.6. Assessment of Signal Peptide Secretion Function

The functionality of effector signal peptides was assessed using the yeast signal sequence trap system [1,19,20]. Primers for the plasmid construction are detailed in Supplementary Table S1. Recombinant pSUC2 vector constructs were introduced into the yeast strain YTK12, which were then cultured on CMD-W media (PM3510, Coollaber, Beijing, China). To evaluate invertase secretion, positive clones were grown on YPRAA medium (PM3011, Coollaber, Beijing, China). Transformants carrying pSUC2-Avr1bSP served as the positive control, while the empty pSUC2 vector acted as the negative control.

## 3. Results

### 3.1. Comprehensive Secreted Proteins Prediction Based on Bioinformatics Software

**Figure 1:**
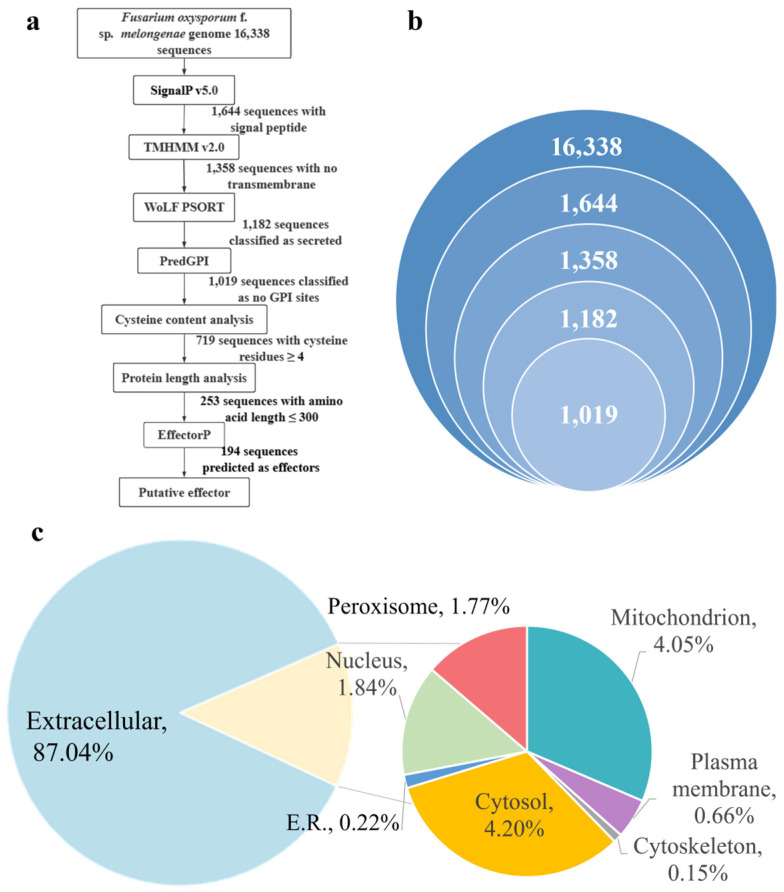
Illustration of putative effector prediction in Fom. (**a**) Workflow for the prediction of putative effectors in Fom. (**b**) The number of secreted proteins screened at each step. (**c**) Subcellular localization of 1019 secreted effectors in Fom predicated by WoLF PSORT.

The *F. oxysporum* f. sp. *melongenae* (Fom) genome encodes 16,338 proteins in total and was used for predicting secreted proteins and CSEPs (Figure 1a). The identification of the secreted proteins was based on distinct characteristics: (I) they possess N-terminal signal peptides, (II) they exhibit predicted localization signals that direct protein delivery extracellularly, and (III) they lack glycosylphosphatidylinositol (GPI) anchor sites. SignalP is the leading online tool for predicting signal peptides in amino acid sequences, capable of identifying potential cleavage sites. In the initial analysis, 1644 proteins with N-terminal signal peptides were recognized, accounting for 10.06% of the total proteins. Further, out of 1644 proteins, 1358 were predicted to contain no transmembrane domain using TMHMM 2.0. Using WoLF PSORT, 1182 protein sequences were predicted to contain extracellularly excreted signal peptides, and 176 proteins were predicted to localize at the cytosol, nucleus, mitochondrion, and others (Figure 1b,c). PredGPI predicted 1019 extracellularly secreted protein sequences as lacking transmembrane structural domains. Out of these, 163 sequences that contained GPI-anchored loci were subsequently excluded. Finally, these 1019 protein sequences were thus considered the candidate secreted proteins (Figure 1).

### 3.2. CAZymes Analysis of Secreted Proteins

**Figure 2:**
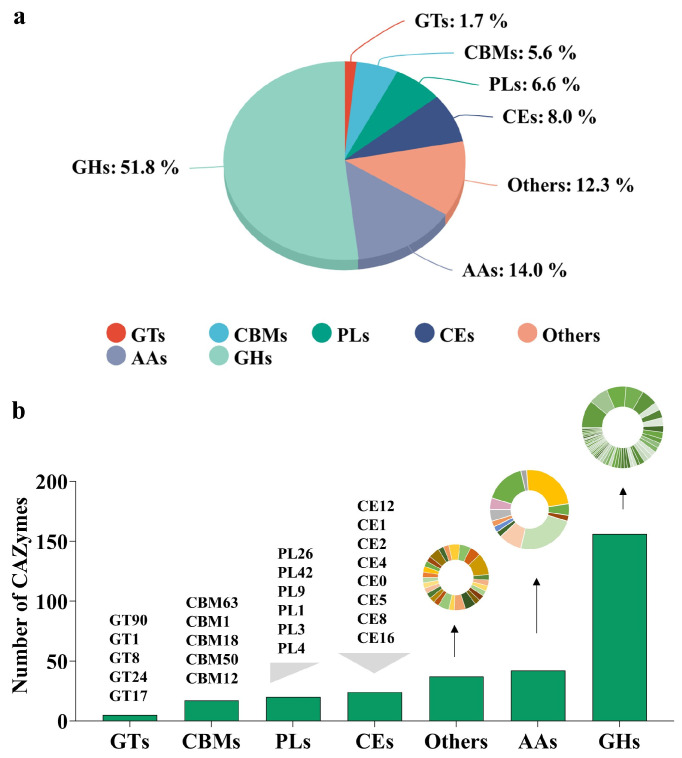
Composition of the subfamilies of CAZymes in Fom. (**a**) The proportion of each subfamily in Fom. (**b**) The number of CAZymes in each subfamily in Fom.

Carbohydrate-active enzymes (CAZymes) are crucial to the pathogenic mechanisms of various organisms. They facilitate the synthesis and degradation of carbohydrates and glycoconjugates, which are essential components of biological functions [21]. CAZymes are classified into six families, including glycoside hydrolases (GHs), glycosyltransferases (GTs), carbohydrate esterases (CEs), carbohydrate binding modules (CBMs), polysaccharide lyases (PLs), and auxiliary module enzymes (AAs) [22]. Among a total of 1019 secreted proteins, 301 proteins were identified as CAZymes; among these CAZymes, the distribution and proportions of the proteins across the six subfamilies were as follows: GHs (156 proteins, 51.82%), AAs (42 proteins, 13.95%), PLs (20 proteins, 6.64%), CEs (24 proteins, 7.97%), CBMs (17 proteins, 5.65%), and GTs (5 proteins, 1.66%). Furthermore, there were 37 proteins belonging to complex CAZymes, accounting for 5.65% of the total secreted proteins (Figure 2a). These categories of proteins exhibit multifunctional enzymatic properties, suggesting their diverse roles in the infection process of Fom.

The cell wall of plants serves as the main defense against pathogen intrusion, prompting pathogens to release a range of enzymes that degrade the cell wall to bypass this defense. The plant cell wall is a complex structure mainly composed of pectin, cellulose, hemicellulose, lignin, and various other polysaccharides and proteins. Pectinases, cellulases, and hemicellulases, the main enzymes involved in this degradative process, are mostly found in nine CAZyme families: CE8, PL1, PL2, PL3, PL9, PL10, GH28, GH78, and GH8 [23]. In further analysis of the subfamilies in each CAZymes family (Figure 2b), we found that the GH family had the most diversity. GH43 was the most abundant subfamily with 17 proteins, followed by GH3 (12 proteins), GH16 (12 proteins), GH28 (11 proteins), and GH5 (10 proteins). In the CEs family, the CE5 subfamily were the most abundant with seven proteins. CE0, CE8, CE16, and CE12 all consisted of three proteins each. The PLs family was led by PL1, with a count of seven proteins. Other subfamilies included PL3 (five proteins), PL4 and PL42 (two proteins each), and PL26 and PL9 (one protein each). In the CBMs family, CBM50 had the most proteins (eight proteins), followed by CBM1 (four proteins), CBM63 (three proteins), and CBM12 and CBM18 (one protein each). The GTs family consisted of five subfamilies, including GT1, GT8, GT17, GT24, and GT90, each represented by one protein. For complex CAZymes, the GH/CBM type was the most common with twenty-one proteins, followed by AA/CBM with eight proteins.

### 3.3. Effector Prediction and Conservation Pattern Analysis

**Figure 3:**
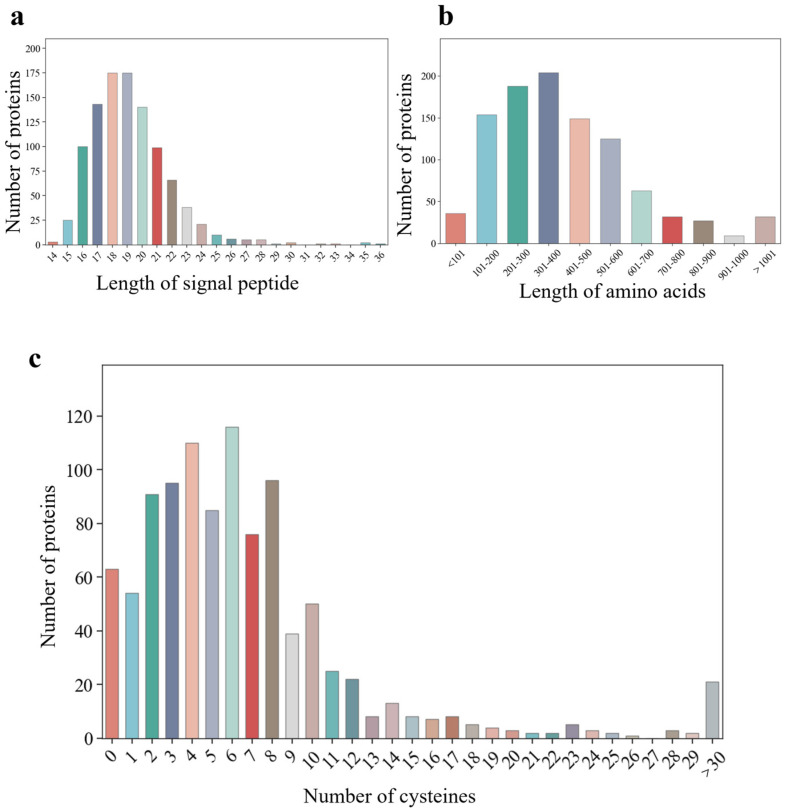
Characterization of secreted effectors in Fom. (**a**) Length of signal peptide of secreted effectors. (**b**) Amino acid length of secreted effectors. (**c**) Number of cysteine residues in secreted effectors.

Signal peptides are short peptide chains that direct the transfer of synthesized proteins to the secretory pathway and are usually 5–30 amino acids in length, which is crucial in the transport and secretion of proteins [24]. A detailed analysis of the signal peptides from 1019 secreted proteins revealed a length range of 14 to 36 amino acids (aa). Most of these peptides, precisely 898 or 88.13% of the total, exhibited lengths within the 16–22 aa range (Figure 3a). Effector proteins, a distinctive group of secreted proteins, are generally distinguished by their low molecular weight and high cysteine content. To investigate these characteristics, we analyzed the amino acid residues and the number of cysteine residues from the 1019 secreted proteins, revealing a notable variation within these secreted proteins. The analysis of amino acid residues showed that most proteins were found within the 100–600 aa range, with the highest proportion (20.02%) falling within 301–400 aa (Figure 3b). The results indicated that secreted proteins exhibited a variable size distribution, likely correlated with their biological functions, which may restrict protein size during cellular transport and functional activities. In terms of cysteine residues, the result showed that most proteins were found within the 2–8 aa range, with proteins containing six cysteines being the most common (Figure 3c). Remarkably, only 63 proteins contained no cysteine, while 54 proteins contained one cysteine. Meanwhile, we documented 91, 95, 110, and 85 effector proteins with two, three, four, and five cysteines, respectively. A search of effector protein motifs using MEME identified three motifs with higher E values, of which Motif 3 was present in multiple secreted proteins. Furthermore, motifs 1–3 were detected in 15, 14, and 25 effector proteins, respectively (Figure S1). Based on the cysteine residue count and protein length (amino acid length ≤ 300) exclusion screens, only 253 proteins were found to meet the characteristics of classical effectors.

EffectorP is a machine-learning method capable of accurately predicting known plastidic ectodomain and cytoplasmic effector proteins in the fungal secretome [25]. Further analysis using the EffectorP software revealed that 194 proteins were predicted as candidate effector proteins. Among these, 54 protein sequences were predicted as cytoplasmic effector proteins, and 140 were predicted as apoplastic effector proteins. Comparing the 194 CSEPs using the PHI database, 192 protein sequences were functionally annotated. Interestingly, 136 protein sequences were predicted to decrease pathogen virulence upon deletion, whereas 46 protein sequences did not affect pathogenicity (Table 1). Seven protein sequences were classified as effectors, and three protein sequences were of the virulence-enhancing type, hypothesized to contribute to pathogenicity.

### 3.4. Comparative Analysis of Classical Secreted and Effector Proteins of Fom and Other Fusarium *spp.*

**Figure 4:**
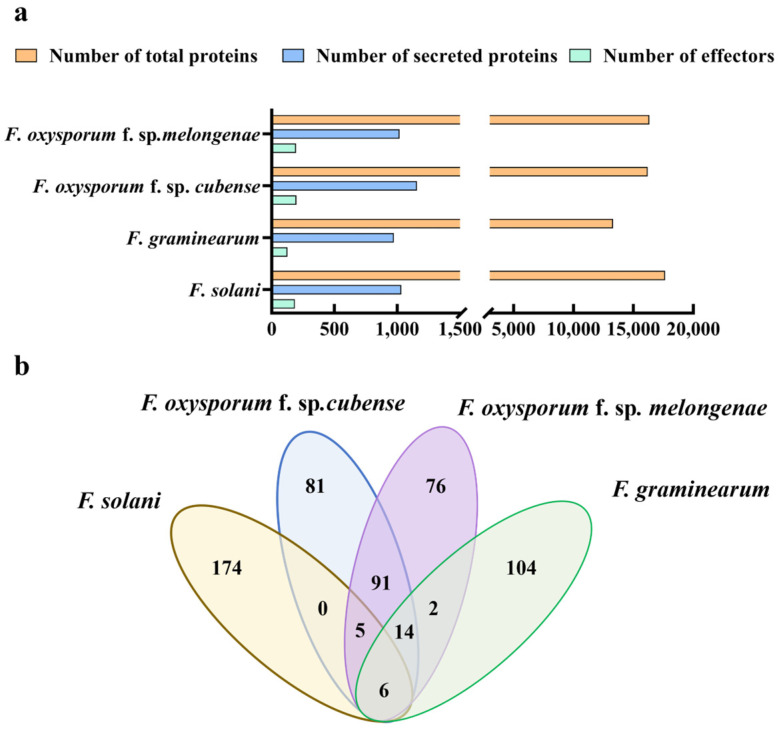
Comparison of secreted proteins and effectors among Fom and three other Fusarium pathogens. (**a**) Number of secreted proteins and effectors in *F. solani*, *F. graminearum*, *F. oxysporum* f. sp. *Cubense*, and Fom. (**b**) Number of conserved core effectors across the four *Fusarium* species.

To investigate the differences in secreted proteins and effector proteins among different pathogenic Fusarium species, the amounts of classical secreted proteins and effector proteins in four species—*F. solani* (Fs), *F. graminearum* (Fg), *F. oxysporum* f. sp. *cubense* (Foc), and Fom—were analyzed using consistent analytical methods and parameters. A comparative assessment of classical secreted proteins and effector proteins among these species was conducted. The results are shown in Figure 4. The Fs genome encodes 17,654 proteins, of which 1032 are predicted classical secreted proteins and 185 are CSEPs; the Fg genome encodes 13,312 proteins, of which 973 are predicted classical secreted proteins and 126 are CSEPs; the Fom genome 16,338 proteins, including 1019 secreted proteins and 194 CSEPs; and the Foc genome encodes 16,202 proteins, with 1158 secreted proteins and 197 effectors (Figure 4a). These findings indicate no correlation between the number of secreted proteins, the number of effectors, and the total number of genetically encoded proteins. Variations in the counts of these proteins among different pathogenic Fusarium species may be related to the host species, infestation strategies, and levels of pathogenicity. Additionally, a comparative analysis of the effector proteins across the four Fusarium species revealed those sharing over a 90% similarity, which were further examined through Venn analysis. The findings revealed the presence of 116 highly conserved effector proteins in both Foc and Fom, another set of 22 highly conserved effector proteins in Fg and Fom, and 10 highly conserved effector proteins in Fs and Fom (Figure 4b). Additionally, six effector proteins exhibited high conservation among all four Fusarium species. It is hypothesized that these six effector proteins have remained highly conserved throughout the evolutionary history of Fusarium.

### 3.5. Analysis of Secreted Proteins Based on Label-Free Proteomic in Fom

**Figure 5:**
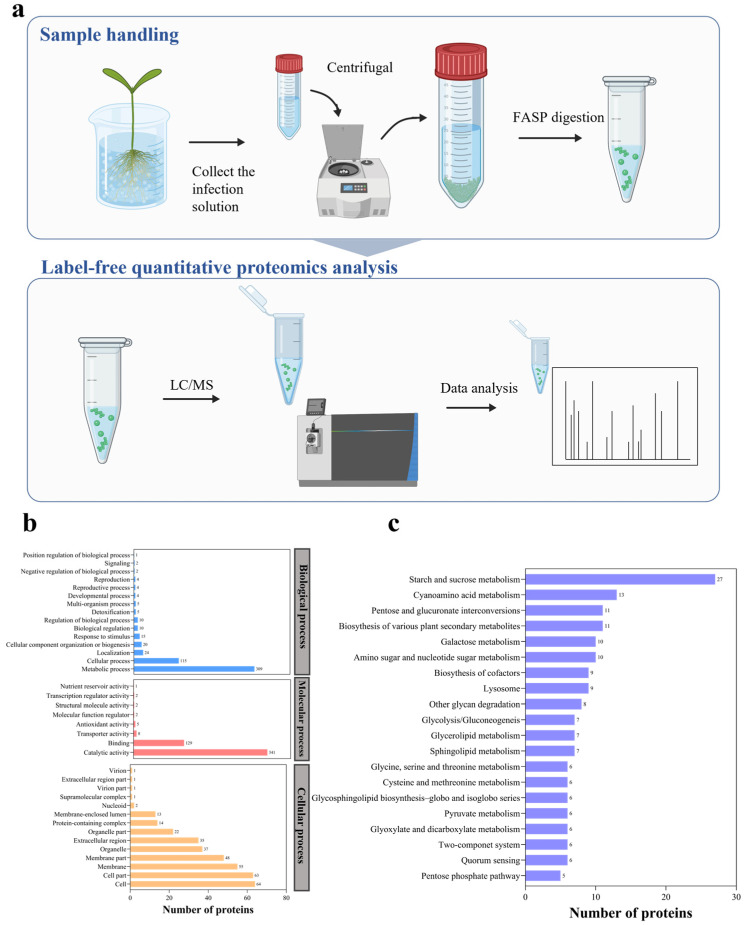
Analysis of the secreted proteins of Fom under induced conditions by label-free quantitative proteomics. (**a**) The process of LC-MS analysis. (**b**) GO analysis of the label-free quantitative proteomics-identified proteins. (**c**) KEGG analysis of the label-free quantitative proteomics-identified proteins.

Mass spectrometry-based label-free quantitative proteomics was utilized to analyze the secreted proteins of Fom under induced conditions (Figure 5a). The secreted proteins of Fom were extracted from eggplant tissue induction medium for 5 d by the acetone precipitation method. A total of 563 proteins were identified, and a gene ontology (GO) enrichment analysis was performed. The identified proteins were mainly associated with metabolic processes, single-organism processes, and localization (Figure 5b). In terms of biological processes, the CSEPs could be classified into 15 categories and were mostly related to metabolic processes and cellular processes. Under the category of molecular processes, the identified proteins were mainly associated with catalytic activity and binding. Under the category of cellular processes, these proteins were mostly related to cellular structure, membrane structure, and organelles.

KEGG enrichment analysis is a critical tool that aids in understanding the biological function of proteins. A KEGG enrichment analysis revealed that among the top 20 pathways, the ‘starch and sucrose metabolism’ pathway was enriched with the most proteins, tallying 27 proteins (Figure 5c). This was followed by ‘cyanoamino acid metabolism’ with 13 proteins (2.31%). Both the ‘pentose and glucuronate interconversions’ and ‘biosynthesis of various plant secondaries’ pathways contained 11 proteins, representing 1.95% each. Other enriched pathways included ‘lysosome’, ‘galactose metabolism’, ‘biosynthesis of cofactors’, and ‘amino sugar and nucleotide interconversions’ (Figure 5c).

**Figure 6:**
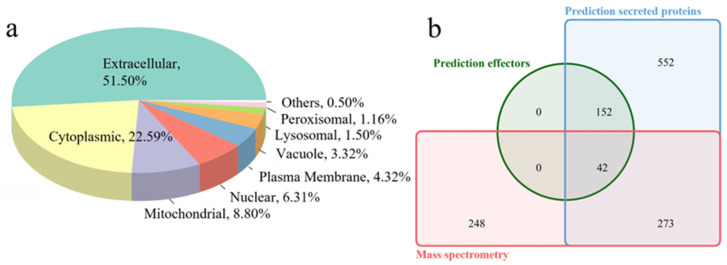
Subcellular localization and comparison analysis of label-free quantitative proteomics-identified proteins. (**a**) Subcellular localization of label-free quantitative proteomics-identified proteins predicated by CELLO. (**b**) Venn diagram showing the extent of overlap between predicted secreted proteins, effectors, and label-free quantitative proteomics-identified proteins.

We also used the CELLO website to predict the subcellular compartment of the 563 identified proteins and discovered that most proteins localized to the extracellular region (51.50%), cytoplasm (22.59%), and mitochondria (8.80%) (Figure 6a). To further validate whether the proteins identified through label-free quantitative proteomics were secretory proteins, we compared them with previously predicted secreted proteins and effectors. Among the 563 proteins, 315 were classified as classical secretory proteins, while 42 were predicted to function as effectors (Figure 6b). Additionally, 72 proteins were identified as non-secretory.

### 3.6. CSEPs Suppressed Programmed Cell Death (PCD) Triggered by BAX in N. benthamiana

**Figure 7:**
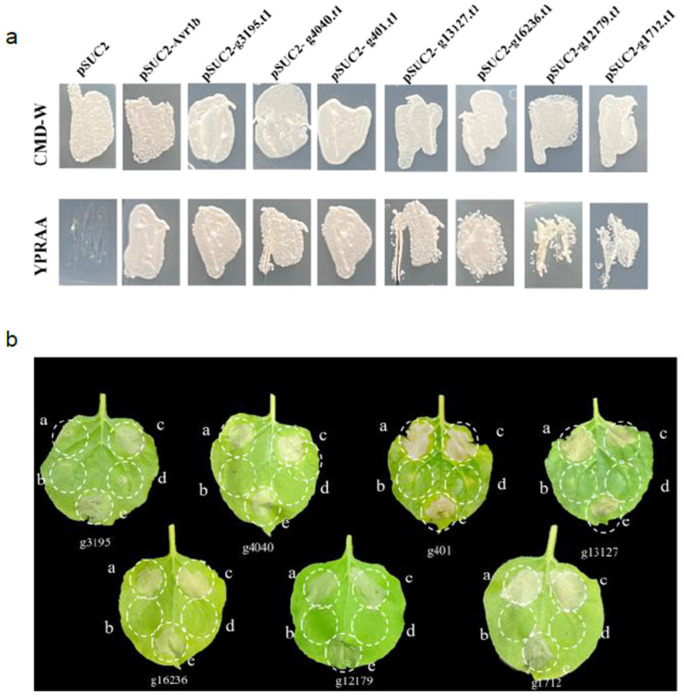
The functional verification of CSEPs. (**a**) Functional validation of the CSEP signal peptides using yeast invertase secretion assays. The signal peptide was fused into the pSUC2 vector and transformed into the yeast YTK12 strain. The predicted signal peptide of pSUC2-Avr1b was used as the positive control, and YTK12 carrying the pSUC2 vector was used as the negative control. (**b**) BAX-triggered cell death in *N. benthamiana* is suppressed by CSEPs: a, CSEP + BAX; b, CSEP; c, GFP + BAX; d, GFP, as negative control; e, BAX, as positive control.

To verify the secretory functions of these forty-two CSEPs, seven out of these CSEPs were chosen to perform a yeast invertase secretion assay. The strains transformed with the pSUC2 vector and Avr1b signal peptide were used as negative controls and positive controls, respectively. The results indicated that all seven CSEPs were capable of growth on YPRAA medium (Figure 7a). This suggests that the signal peptides of these CSEPs rescued the YTK12 strain’s sucrose invertase gene defect, thereby enabling invertase secretion. This result confirmed that these CSEPs had secretory functions.

### 3.7. Validation of the Signal Peptides of CSEPs

To further elucidate the functions of these seven CSEPs in plant immunity, a virus-based transient expression system was conducted. A. tumefaciens transformed with GFP served as a negative control, while those transformed with BAX was employed as a positive control. The result showed that the CSEP g3195 could suppress BAX-triggered programmed cell death in N. benthamiana, whereas other CSEPs neither induced cell death nor suppressed BAX-induced cell death. To further study the function of g3195, we constructed a vector of g3195 without a signal peptide. Our results showed that g3195^△SP^ could not inhibit BAX-induced cell death (Figure S2). This suggests that g3195^△SP^ is unlikely to be secreted into the cellular extracellular space, and hence, its expression would not suppress BAX-triggered programmed cell death. Based on these findings, we hypothesized that g3195 may play an important role in suppressing plant immune responses during Fom infection processes. 

## 4. Discussion

Secreted effector proteins are critical virulence or avirulence factors that enable the pathogenic fungal invasion of hosts, which are secreted extracellularly to induce plant death or trigger plant immune responses. The widespread adoption of genome-wide sequencing technologies has facilitated the acquisition and analysis of diverse pathogen genomes, including those of *Colletorichum gloeosprioides*, *Verticillium dahliae*, *Rhizoctonia solani*, *Magnaporthe oryzae*, *F. graminearum*, and *F. sacchari*. Bioinformatics is the predominant method employed for predicting effectors, relying on relatively broad criteria primarily focused on the presence of a secretion signal. Previous studies have demonstrated that various secreted proteins are linked to the virulence of pathogenic fungi. For example, BcXyl1 [26], BcCrh1 [27], BcIEB1 [28], and BcXyg3 [29] in *Botrytis cinerea* have been shown to trigger diverse responses, such as plant cell apoptosis, callose deposition, and reactive oxygen species (ROS) burst; Bas3, Bas4, Bas83, Bas113, and Bas170 in *M. oryzae* suppress the plant immune response by disrupting the rice salicylic acid and ethylene pathways, suppressing the immune response to chitin, and reducing the burst of reactive oxygen species, among other responses [30]; AvrM14, the Nudix hydrolase effector in *Melampsora lini*, interferes with plant immune responses by inhibiting the production of ROS production and degrading mRNA [31]. Previous studies have verified that many secreted proteins are associated with virulence in *Fusarium* [32], such as FolAsp [33], FGL1 [34], FoEG1 [35], Fosp9 [36], and FoAPY [37]. These effector proteins vary in their morphological structures and functions. Based on this, we reasonably speculate that there may be specific virulence factors in different formae specialis. These specific factors are likely to be one of the reasons for the differences in the host ranges of different formae specialis of pathogens. They play a crucial role in the specific interaction between pathogens and hosts, profoundly influencing the process of pathogen selection and infection of the hosts.

To elucidate the molecular mechanism underlying the interactions between Fom and eggplant, we employed a variety of computational prediction methods, such as SignalP, WoLF PSORT, TMHMM, and EffectorP to predict CSEPs. SignalP v5.0 serves as a reliable tool for screening protein sequences, effectively narrowing down the pool of potential secreted proteins by integrating multiple artificial neural networks to distinguish SP/non-SP. WoLF PSORT facilitates large-scale predictions of protein localization, providing essential insights into the functional sites of plant intracellular effectors. Additionally, TMHMM employs hidden Markov models to predict transmembrane domains in proteins. Subsequently, PredGPI was utilized to identify protein sequences lacking GPI anchor sites. The integration of these bioinformatics tools significantly refined our predictions of CSEPs, resulting in the identification of 1019 proteins consistent with classical secretory protein characteristics, which accounted 6.24% of the total genome proteins. Cysteine content is a common criterion for identifying candidate apoplastic effector proteins, as many cysteine residues in fungi are thought to form the intramolecular disulfide bonds that are essential for stability and function in the protease-rich environment of the apoplast. In accordance with the characteristics of CSEPs, which typically comprise fewer than 300 amino acids, we manually excluded sequences shorter than 30aa and those containing more than four cysteine residues for further analysis. Consequently, we identified 253 protein sequences, accounting for 24.83% of the secreted proteome. EffectorP is a prediction program based on machine training to predict fungal effectors according to a series of properties of proteins. We submitted the protein sequences for further prediction. Ultimately, a total of 194 protein sequences were deemed final CSEPs based on the predictions generated. We submitted these 194 protein sequences to the PHI database for functional annotations, resulting in 192 annotated proteins. Most of these proteins were associated with reduced virulence or unaffected pathogenicity, while three proteins were linked to increased virulence. Further analysis of these three effector proteins revealed that g9683.t1 exhibited similarity to BAS4 in *M. oryzae*, previously identified as a biotrophic-associated secreted protein and highly expressed during the infection stage, playing an important role in the pathogen–rice interaction process [38]; g5832.t1 was analogous to Sm1 in *Trichoderma virens*, which enhances plant resistance by activating plant defense responses [39]; and g4066.t1 was found to be similar to BcFET1 in *B. cinerea*, which reduced cellular iron content, negatively impacting growth and development [40].

Proteomic analysis has become an essential methodology for investigating plant–pathogen interactions, particularly concerning the secreted proteins of plant pathogenic fungi. As noted by Gonzalez-Fernandez and Jorrín-Novo, the application of these techniques is on the rise [41]. A key advantage of proteomic methodologies is their capacity to identify proteins integral to host–microbe interactions. Liquid chromatography–tandem mass spectrometry (LC-MS/MS) serves as a cornerstone of proteomic analysis, functioning effectively in both gel-based and gel-free formats. Numerous studies utilizing LC-MS/MS have yielded significant insights. For instance, research on *Macrophomina. phaseolina* revealed 117 proteins present in its secretome [42]. Additionally, a study focusing on *F. oxysporum* f. sp. *lycopersici* identified 50 potential secreted proteins, with specific acetylation sites elucidated through LC-MS/MS analysis [43]. In our study, a total of 563 proteins were detected within the secretome of Fom. These results highlight the efficacy of proteomic methods, particularly LC-MS/MS, in identifying specific proteins released by plant pathogenic fungi. This information enhances our understanding of host–pathogen interactions and may facilitate the development of improved management strategies for pathogenic fungi.

CAZymes constitute a significant portion of pathogen’s secreted proteome and function as virulence factors by degrading the host cell wall, thereby facilitating pathogen invasion and colonization in plants. Utilizing the dbCAN3 database, we identified a total of 301 CAZymes proteins, accounting for 29.53% of the total secreted proteins. These proteins were classified into six families: GHs, GTs, CEs, CBMs, PLs, and AAs. Notably, the GH family comprised the largest subset, accounting for 51.83% of the total CAZymes identified. Previous studies have demonstrated that GHs are widely present in diverse fungi, bacteria, and oomycetes, exhibiting the activities of cellulase (GH5, GH6, GH7, GH9, and GH12), chitinase (GH18 and GH85), xylanase (GH10, GH11, and GH30), and β-1,3-1,4-glucanase (GH1, GH3). These enzymes effectively degrade the complex polysaccharide structure of plant cell walls and play a crucial role in both triggering plant immunity and facilitating pathogenic infection [44,45]. In comparison to other formae speciales of *F. oxysporum*, *F. oxysporum* f. sp. *albedinis* (Foa) Foa133 and Foa9 possess 74 and 90 glycoside hydrolases (GHs), respectively [46]. Notably, the number and variety of GHs in Fom are significantly greater than those in Foa. Meanwhile, *F. oxysporum* f. sp. *ciceris* (Foci) contains 105 GTs [47]. However, only five GTs proteins were identified in Fom, and GTs are completely absent in Foc1 and Foc4 [48]. Plant cell walls are typically rich in lignin, a non-carbohydrate component, and AAs contribute to its degradation. In Fom, the AAs family comprises 42 proteins, accounting for 13.95% of the total CAZymes, and encompasses 15 subfamilies. Among them, AA3 and AA9 are the most prevalent; the AA3 subfamily primarily contains GMC oxidoreductases, including alcohol oxidase, aryl alcohol oxidase, glucose oxidase, fiber disaccharide dehydrogenase, pyranose oxidase, etc., while AA9 mainly degrades polysaccharide monooxygenase [45]. Moreover, much AA1 was found in Fom, which ranked third in abundance after AA3 and AA9. Previous studies indicate that AA1 predominantly functions as a multicopper oxidase characterized by copper-binding motifs including His-Xaa-His-Gly (HXHG), His-Xaa-Xaa-His-Xaa-His (HXXHXH), and His-Cys-His-Xaa(3)-His-Xaa(4)-Met/Leu/Phe (HCHXXXHXXXXM/L/F) [49]. Additionally, the β-α-β dinucleotide binding motif Gly-Xaa-Gly-Xaa-Xaa-Gly-Xaa18-Glu (GXGXXGX18E) present in the GMC oxidoreductase of AA3 underpins substrate interactions, which are critical for the degradation of non-carbohydrate substances [50,51,52]. CEs are enzymes that facilitate the deacylation of esters or amides, such as aspartate esterase (CE4), acetyl xylan esterase (CE1), and feruloyl esterase (CE1, CE6) [53]. Our study reveals that the composition and types of CAZymes subfamilies in Fom differ markedly from those in other strains, likely due to variations in host specificity. Fom infects eggplants, necessitating the degradation of their thicker lignified layer in the roots and stems, which thereby requires an increased presence of cellulases. Consequently, these differences in CAZymes’ profiles may significantly contribute to the successful infection of formae speciales strains in plants.

This study employed the tobacco transient expression system to evaluate the functions of seven CSEPs of Fom. The findings demonstrated that g3195.t1 exerts an inhibitory effect on BAX-induced programmed cell death. However, there are differences in physiological structure and immune response mechanisms between eggplant and *N. benthamiana*, as well as in immune signaling pathways. Therefore, the function of g3195 needs to be further verified using eggplant as the host in subsequent experiments to more accurately study the interaction between eggplant and Fom. GO annotation revealed that this protein possesses hydrolase activity, contains a conserved cutinase domain, and is categorized as a cutinase. The plant cuticle, primarily composed of cutin, wax, and cellulose, serves as the initial defense against fungal infections [54,55]. Plant pathogenic fungi compromise the integrity of plant cell walls through various cell wall-degrading enzymes, facilitating invasion. Cutinase is critical in this process, typically released during the early stages of infection to degrade the cuticle, the plant’s first line of defense [56]. Consequently, the knockout or inhibition of cutinase significantly affects the virulence of plant pathogenic fungi. In *F. sacchari*, the cutinase G-box binding protein FsCGBP is implicated in regulating the MAPK pathway, thus playing a critical role in the growth, development, and virulence of the fungus [57]. Similarly, in *C. truncatum*, *C. gloeosporioides*, *Botryosphaeria dothidea*, and *Arthrinium phaeospermum*, the lack of cutinase has been associated with reduced virulence [58,59,60,61]. A previous study demonstrated that the application of enzyme inhibitors to block cutinase activity effectively prevents *F. solani* from infecting plants [62]. Additionally, *Sclerotinia sclerotiorum* secretes significant amounts of cutinase during infection, compromising the plant cuticle, with SsCut1 promoting its virulence by enhancing cutinase activity [63]. Nevertheless, the role of g3195 in the interaction between pathogens and the plant immune response remains to be elucidated. In subsequent research, we will conduct further verification of the function of g3195, identify the changes in its expression level during the pathogen infection process, search for interacting target proteins in eggplant to obtain target genes for breeding resistance, and thus provide a new direction for eggplant resistance breeding.

## 5. Conclusions

This study has achieved substantial progress in comprehending the pathogenic mechanism of Fom through a systematic analysis of its secreted proteins. Out of 1019 identified secreted proteins in Fom, 194 have been recognized as CSEPs, thereby considerably enhancing our understanding of potential effectors in this fungal species. The annotation of various secreted proteins suggests their prospective roles in facilitating Fom to invade host plants. Transient expression assays conducted in tobacco confirmed the involvement of the candidate effector g3195 in the pathogenic process, supporting its hypothesized critical role in pathogenicity. Future functional investigations of these secreted proteins linked to pathogenicity may yield new insights into Fom and suggest potential strategies for infection management. Overall, this study offers significant clues for continuing to explore the intricate pathogenic mechanisms underlying Fom.

## Figures and Tables

**Table 1:** Function annotation of 192 CSEPs in Fom.

Type	Number of Proteins
Reduced virulence	136
Unaffected pathogenicity	46
Effector	7
Increased virulence (hypervirulence)	3

## Data Availability

Data will be made available on request.

## Supplementary Materials

The following supporting information can be downloaded at: https://www.mdpi.com/article/10.3390/jof10120828/s1, Table S1: Primer used in this study; Figure S1: De novo prediction of secreted proteins; Figure S2: BAX-triggered cell death in *N. benthamiana* is suppressed by g3195.
