# Supplementary material for: Comprehensive Genomic and Proteomic Analysis Identifies Effectors of Fusarium oxysporum f. sp. melongenae
Source: J Fungi (Basel). 2024 Nov 28;10(12):828. doi: 10.3390/jof10120828 (PMC11678241; doi:10.3390/jof10120828)
Supplement: Supplementary file 1 [file jof-10-00828-s001.zip › Supplementary material S2.pdf]

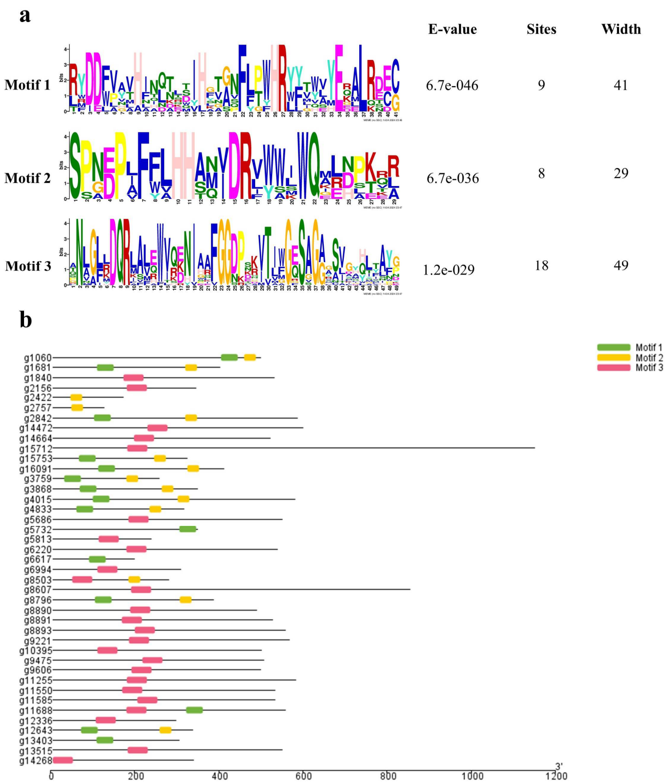

Figure S1. De novo prediction of secreted proteins. (**a**) The prediction of three new motifs of secreted proteins by MEME. (**b**) The motifs location of secreted proteins.

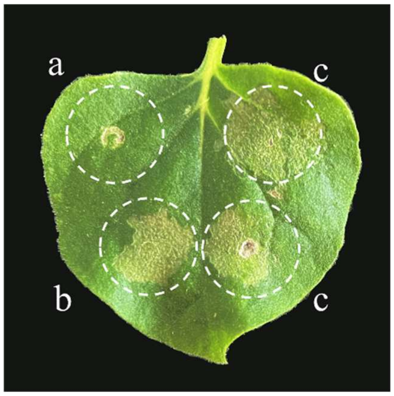

Figure S2. Transient expression of g3195 suppresses cell death triggered by Bax. a, g3195 + Bax; b, g3195 $^{\triangle SP}$ + Bax; c, Bax.
